# A Rare Case of Successful Management of Postpartum COVID-19-Related Coagulopathy

**DOI:** 10.7759/cureus.50986

**Published:** 2023-12-23

**Authors:** Ritesh R Joshi, Rumi Bhattacharya, Shilpa Sapre

**Affiliations:** 1 Obstetrics and Gynaecology, Pramukhswami Medical College, Karamsad, IND; 2 Obstetrics and Gynaecology, Shree Krishna Hospital, Bhaikaka University, Karamsad, IND

**Keywords:** pe thrombolysis, hypercoagulation, ct pulmonary angiogram (ctpa), high-risk pregnancy, acute pulmonary embolism

## Abstract

Pulmonary embolism, while a rare and life-threatening condition, is increasingly recognized in the context of pregnancy, particularly when compounded by infections such as coronavirus disease 2019 (COVID-19). This case report describes a 34-year-old pregnant woman, at 32 weeks of gestation, who developed COVID-19-related coagulopathy despite prophylactic treatment with low molecular weight heparin (LMWH). Admitted with symptoms of breathlessness, fever, and cough, she was diagnosed with COVID-19 and subsequently developed pulmonary embolism. This development was particularly unusual given her ongoing LMWH therapy. Diagnosis in such cases relies heavily on clinical assessment, evaluation of risk factors, and diagnostic tools, with computed tomography pulmonary angiography being pivotal for confirmation. This case highlights the complexities involved in managing COVID-19 among pregnant patients, especially the increased risk and diagnostic challenges associated with thromboembolic disorders. The successful resolution of the case, attributed to a multidisciplinary team's timely intervention and coordinated approach, emphasizes the critical need for prompt and individualized treatment strategies in similar clinical scenarios.

## Introduction

Pregnancy is a hypercoagulable state, increasing the risk of venous and arterial thromboembolic disorders [1]. Conditions like lung infections, prolonged hospitalization, and surgery can exacerbate this risk. Since the initial coronavirus disease 2019 (COVID-19) outbreak in Wuhan, China, the global community has been grappling with its effects. COVID-19, beyond causing flu-like symptoms, can also lead to hypercoagulation, blood stasis, and endothelial damage, potentially resulting in life-threatening thromboembolic disorders [2]. Consequently, prophylactic anticoagulant therapy is a primary treatment approach to prevent such complications [3]. This case report describes the occurrence of COVID-19-related coagulopathy in a postpartum woman despite prophylactic anticoagulant use. Timely diagnosis and treatment of pulmonary embolism were crucial in achieving successful maternal and fetal outcomes.

## Case presentation

A 34-year-old pregnant woman with a history of one vaginal delivery and no comorbidities at 32 weeks' gestation, presented to our emergency department. Over the span of seven days, she described a worsening condition involving shortness of breath, fever, cough, and exhaustion. Initially, she self-medicated at home, largely due to COVID-19 concerns, and later confirmed the diagnosis on COVID-19 reverse transcriptase-polymerase chain reaction (RT-PCR). Her current and previous pregnancies had been uneventful, with normal antenatal check-ups and scans at a private hospital.

**Figure 1:**
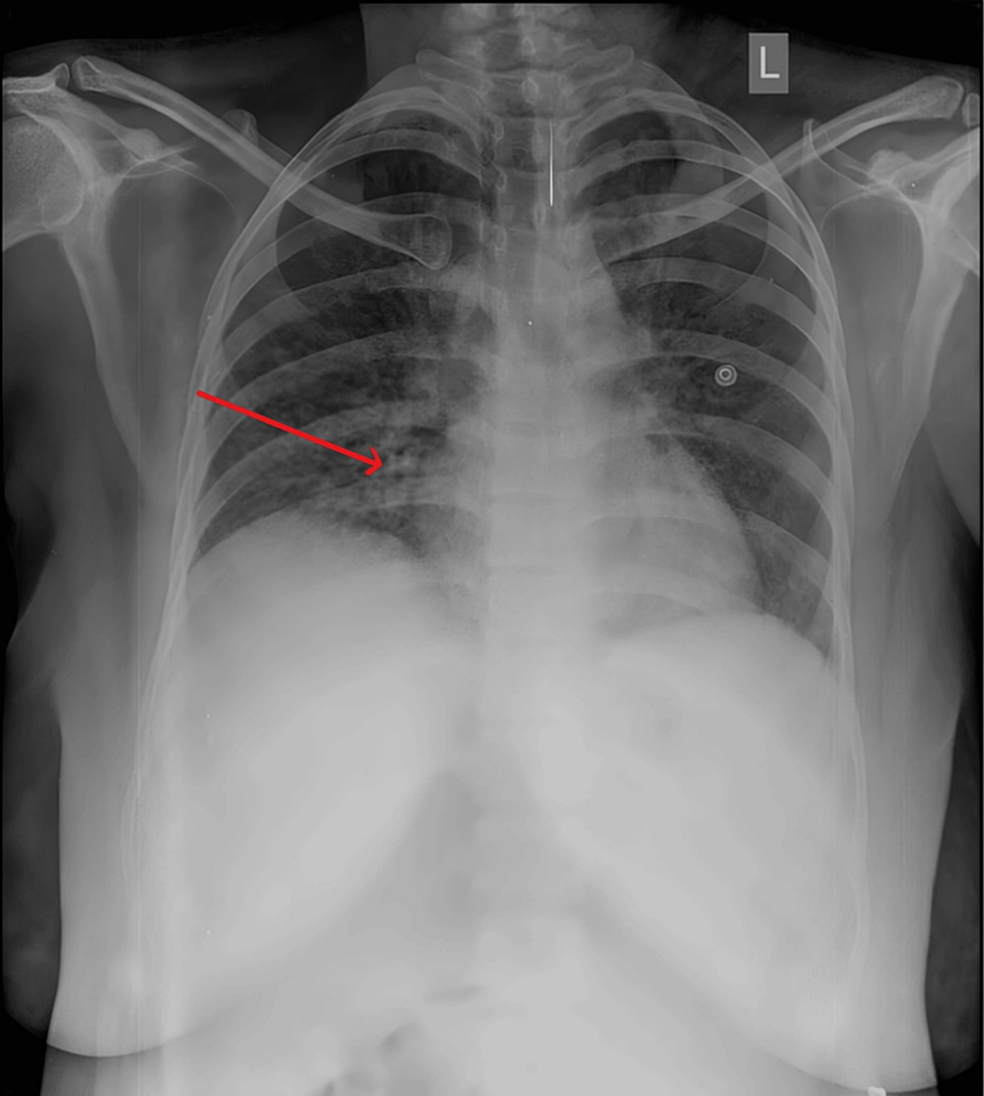
Chest X-ray suggestive of early signs of pneumonia.

Upon admission, the patient was conscious, with a pulse of 120 bpm, blood pressure of 100/60 mmHg, and an oxygen saturation of 80% on room air. Lung auscultation revealed bilateral crackles. Abdominal examination showed a 32-week-sized uterus with fetal heart sounds present. Based on the symptoms observed, there was a suspicion that the individual might have contracted COVID-19 or could be experiencing other conditions like myocardial infarction, sepsis, or myocardiopathy. Consequently, the patient was admitted to the intensive care unit (ICU) and was placed under the care of a multidisciplinary team consisting of an Obstetrician, Intensivist, Pulmonologist, and Neonatologist. The investigation consisted of a full blood count, C-reactive protein (CRP), renal function test, liver function test, electrocardiogram (ECG), coagulation profile, COVID-19 RT-PCR and chest X-ray, which confirmed atypical viral pneumonia due to COVID-19 (Figure 1). Initial treatment included a non-rebreather mask for oxygen, broad-spectrum antibiotics, steroids, prophylactic low molecular weight heparin (LMWH) as an anticoagulant, and other supportive medications.

Facing refractory hypoxia and escalating respiratory distress, the team decided to intubate the patient and perform an emergency lower segment cesarean section at 33 weeks of gestation. A healthy male infant weighing 2.3 kg was delivered and placed in the neonatal intensive care unit for observation. His COVID-19 test was negative, and he was discharged on the seventh postnatal day. The mother remained in the ICU on mechanical ventilation and received COVID-19 treatments.

**Figure 2:**
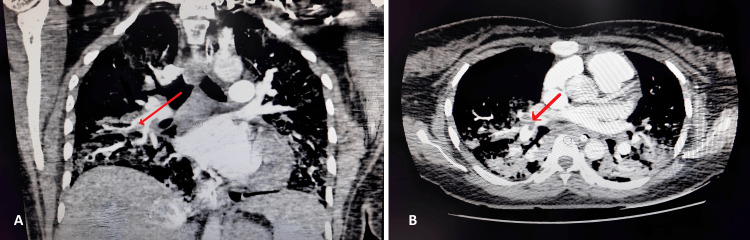
Coronal (A) and axial (B) CTPA confirmed acute thromboembolism in the lumen of the right interlobar, right middle lobar, and right lower lobar pulmonary artery. CTPA: computed tomography pulmonary angiography.

On the seventh postop day, the patient's blood profile showed significantly elevated D-dimer (10,534 ng/ml) and activated partial thromboplastin time (APTT) levels (37.4 seconds) alongside persistent ventilatory support needs. This prompted the team to consider differential diagnoses like cardiac failure or pulmonary embolism. However, that can also be observed in patients who have undergone surgery. Subsequent two-dimensional echocardiography and computed tomography pulmonary angiography confirmed acute thromboembolism in the right interlobar, middle lobar, and lower lobar pulmonary arteries (Figure 2).

After discussing the benefits and risks of thrombolytic therapy, written consent was obtained from the patient's next of kin. Intravenous alteplase (50 mg over two hours, followed by a 25-mg/hour infusion) was administered. Positive signs of recovery were noted, including reduced oxygen requirements and decreasing D-dimer levels.

The patient's overall condition gradually improved, allowing for weaning off ventilator support. She was then transferred to a ward with minimal supplemental oxygen via nasal prongs. Alongside medication, chest physiotherapy and limb mobilization were provided. Her oxygen dependence decreased significantly, and she maintained satisfactory oxygen saturation on room air. Repeat tests showed reduced D-dimer, APTT, and CRP levels (Table 1). She was discharged with stable hemodynamics and received a three-month prescription for anticoagulation treatment, and her postnatal follow-up appointments proceeded without any noteworthy incidents.

**Table 1:** Laboratory values over time Hb: hemoglobin; TLC: total leukocyte count; CRP: C-reactive protein; PT: prothrombin time; INR: international normalized ratio; APTT: activated partial thromboplastin time.

Laboratory Investigation	Date of Analysis							
	5-April	8-April	14-April	20-April	21-April	22-April	28-April	2-May
Hb (g/dL)	11.3	10.8	11.8	11.2	11.8	11.4	12.3	11
TLC (x10^9^/µL)	9.3	11	11.6	10.6	15.3	12.3	12.9	10
CRP (mg/L)	83	60	65.2	99	92	75	42	24
D-dimer (ng/mL)	1882	1352	1052	4042	10,534	7685	3289	1800
PT (seconds)	11.2	11.4	13	15	16	13	12	11.1
INR	0.99	1.01	1.02	1.52	1.56	1.2	1.01	0.98
APTT (seconds)	29.9	30	29	32	37.4	35.2	32	29.6

## Discussion

A significant proportion of pregnant women with COVID-19 remain asymptomatic. In those who are symptomatic, the severity can vary from mild to severe [4]. According to a study by the Centers for Disease Control and Prevention, 31% of antenatal patients hospitalized with COVID-19 are either moderately or critically ill [5].

The incidence of COVID-19-related coagulopathy in non-pregnant patients admitted to the ICU is estimated to be between 25% and 30% [6]. The term "COVID-19-related coagulopathy" has been coined to differentiate the venous and arterial thromboembolic disorders associated with COVID-19 from other well-known thrombotic disorders, such as antiphospholipid syndrome, disseminated intravascular coagulation, hemophagocytic syndrome, and heparin-induced thrombocytopenia [7]. This condition is characterized by typical changes in the coagulation profile including D-dimer, APTT, and fibrinogen levels [7].

In our case, the patient exhibited elevated coagulation markers consistent with COVID-19-related coagulopathy. D-dimer levels can rise to five times the normal range in patients with COVID-19, serving as an indirect indicator of thrombus formation and increased mortality risk [7]. Prophylactic administration of LMWH reduces the risk of thromboembolic disorders in COVID-19 patients. However, our patient developed a pulmonary embolism despite receiving prophylactic LMWH from the time of hospital admission.

The risk factors for venous thromboembolism (VTE) in our patient were limited to pregnancy, COVID-19 infection, and surgery, with no other underlying factors like high body mass index or a personal or family history of VTE. Patients with pulmonary embolism often present with breathlessness, dyspnea, and tachypnea. However, diagnosing pulmonary embolism can be challenging in mechanically ventilated patients, where persistent ventilatory needs and a sharp increase in D-dimer levels can serve as indirect markers of thrombus formation.

## Conclusions

The distinctive aspect of our case report lies in how we managed the COVID-19-related coagulopathy in a patient receiving mechanical ventilation. The involvement of a multidisciplinary team, prompt diagnosis, and early intervention were the key to our success. By sharing this case, we aim to provide insights that could assist frontline healthcare workers in managing patients with a high suspicion of thromboembolic disorders in the context of COVID-19.
